# Natriuretic Peptide-Based Novel Therapeutics: Long Journeys of Drug Developments Optimized for Disease States

**DOI:** 10.3390/biology11060859

**Published:** 2022-06-03

**Authors:** Tomoko Ichiki, Atsushi Jinno, Yoshihisa Tsuji

**Affiliations:** Department of General Medicine, Sapporo Medical University, S1 W17, Sapporo 060-8556, Japan; atsushi.jinno@sapmed.ac.jp (A.J.); ytsuji@sapmed.ac.jp (Y.T.)

**Keywords:** natriuretic peptides, heart failure, hypertension, dwarfism

## Abstract

**Simple Summary:**

Natriuretic peptides are endogenous hormones produced in the heart and vascular endothelium, and they enable cardiorenal protective actions or bone growth via cGMP stimulation through their receptor guanylyl cyclase receptor A or B. To optimize the drug for each disease state, we must consider drug metabolism, delivery systems, and target receptor(s). This review summarizes attempts to develop novel natriuretic peptide-based therapeutics, including novel designer natriuretic peptides and oral drugs to enhance endogenous natriuretic peptides. We introduce some therapeutics that have been successful in clinical practice, as well as the prospective drug developments in the natriuretic peptide system for disease states.

**Abstract:**

The field of natriuretic peptides (NPs) as an endocrine hormone has been developing since 1979. There are three peptides in humans: atrial natriuretic peptide (ANP) and B-type natriuretic peptide (BNP), which bind to the guanylyl cyclase-A (GC-A) receptor (also called natriuretic peptide receptor-A (NPR-A)), and C-type natriuretic peptide (CNP), which binds to the GC-B receptor (also called the NPR-B) and then synthesizes intracellular cGMP. GC-A receptor stimulation has natriuretic, vasodilatory, cardiorenal protective and anti-renin–angiotensin–aldosterone system actions, and GC-B receptor stimulation can suppress myocardial fibrosis and can activate bone growth before epiphyseal plate closure. These physiological effects are useful as therapeutics for some disease states, such as heart failure, hypertension, and dwarfism. To optimize the therapeutics for each disease state, we must consider drug metabolism, delivery systems, and target receptor(s). We review the cardiac NP system; new designer NPs, such as modified/combined NPs and modified peptides that can bind to not only NP receptors but receptors for other systems; and oral drugs that enhance endogenous NP activity. Finally, we discuss prospective drug discoveries and the development of novel NP therapeutics.

## 1. Introduction

### 1.1. Biology of Natriuretic Peptides

Since 1979, when de Bold reported that protein extracted from heart atrium had a natriuretic effect, the story of natriuretic peptides (NPs) as an endocrine hormone has been developed [1]. Then, biologically functional natriuretic peptides were isolated from 1980 to 1990 [2,3,4]. There are three types of peptides in humans: atrial natriuretic peptide (ANP) and B-type natriuretic peptide (BNP), which bind to the membrane-bound particulate guanylyl cyclase (GC)-A receptor (also called the natriuretic peptide receptor [NPR]-A); and C-type natriuretic peptide (CNP) which binds to the membrane-bound particulate GC-B receptor (also called the NPR-B) [5,6,7]. After binding these receptors, NPs have physiological functions via the synthetization of their second messenger, intracellular cyclic guanosine monophosphate (cGMP) [5,6,7].

**Figure 1:**
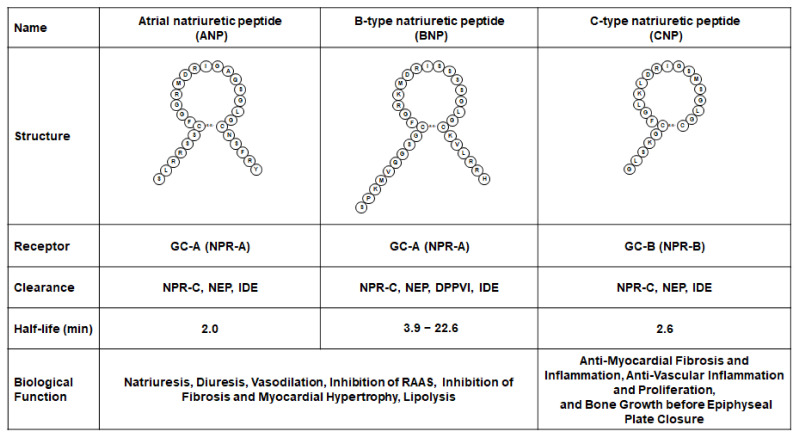
Native natriuretic peptides in human. DPPIV, dipeptidyl peptidase-4; GC-A, guanylyl cyclase-A; GC-B, guanylyl cyclase-B; IDE, insulin degrading enzyme; NEP, neprilysin; NPR-A, natriuretic peptide receptor A; NPR-B, natriuretic peptide receptor B; NPR-C, natriuretic peptide receptor C; RAAS, renin–angiotensin–aldosterone system.

The three native NPs are genetically distinct but share structural similarities [5,6]. Figure 1 illustrates three NPs that consist of a 17 amino acids ring structure created by a disulfide bond joining two cysteine residues with distinct N- and C-terminal extensions (Figure 1). They are produced as preprohormones that are subsequently processed into prohormones via the cleavage of an N-terminal signal peptide. All three NPs are cleared by a non-cyclic GMP-linked receptor, called NP receptor-C (NPR-C), by endocytosis, and they are also degraded by neprilysin, dipeptidyl peptidase-4, or insulin degrading enzyme (Figure 1) [5,6]. The half-life of each peptide is also shown in Figure 1 [5].

GC-A receptor activator, ANP and BNP are released by myocardial stretch, and then GC-A receptor stimulation has such effects as natriuresis, diuresis, vasodilation, the inhibition of the renin–angiotensin–aldosterone system (RAAS), enhanced myocardial relaxation, the inhibition of fibrosis and hypertrophy, and the promotion of cell survival [8,9]. An emerging concept posits that GC-A receptor activation induces lipolysis, promotes browning of white adipocytes, increases mitochondrial oxidative metabolism and fat oxidation in skeletal muscles, and enhances glucose uptake [10,11,12]. In particular, increased adrenal aldosterone synthesis and secretion are seen in heart failure (HF), hypertension (HT), and various cardiovascular diseases [13,14], which is a very important therapeutic target. ANP can suppress aldosterone production in the glomerular layer of the adrenal cortex [15], and showed further inhibition of adrenal aldosterone secretion by angiotensin receptor blockers [16].

The GC-B receptor activator CNP is the most conserved NP across species, and it is thought to mediate its actions through a paracrine or autocrine mechanism [6,17]. GC-B receptor stimulation has such effects as the suppression of myocardial fibrosis, inflammation and contractile dysfunction, anti-proliferative, anti-thrombotic and anti-inflammatory actions on the vascular endothelium together with vasorelaxant, and bone growth before epiphyseal plate closure [6,18].

As cGMP synthesizers, there are not only NPs but nitric oxide (NO) donors. NO binds to intracellular soluble guanylyl cyclase (sGC) and NPs bind to particulate GC receptors, respectively, and increase cGMP formation. The synthesized cGMP is then abrogated by cGMP hydrolysis via phophodiesterases (PDEs) [19]. The expressions of sGC, pGC, or PDEs are highly different in cell types and pathophysiologic conditions, and there is potential crosstalk that would affect intracellular cGMP levels [18,19]. Even as a cGMP synthesizer, the therapeutic effects by particulate GC receptor activators and sGC stimulators seem to be different. For instance, BNP infusion showed better cardiac unloading effects and decreased pulmonary congestion than NO donor nitroglycerin in hospitalized patients with decompensated acute HF [20]. ANP infusion also resulted in the suppression of circulating angiotensin II, aldosterone, and endothelin-1 levels together with anti-LV remodeling effects compared to nitroglycerin infusion in the acute phase of acute myocardial infarction [21]. In this review, we focus on the therapeutic use targeting particulate GC receptors.

### 1.2. Therapeutics of Native Natriuretic Peptides and the Directions

Recombinant human ANP (carperitide) for intravenous injection/infusion in Japan and synthetic human BNP (nesiritide) in the United States (US) have been used as treatments for acute HF [9,22]. Carperitide has been used successfully in Japan for the treatment of acute decompensated HF; proving safe and effective in the COMPASS trial [23] and improved prognosis in the PROTECT trial [24]. Nesiritide was also used for the treatment of acute decompensated HF, however, phase III clinical trial ASCEND-HF did not show an improvement of prognosis by nesiritide compared to a placebo [25], and the Food and Drug Administration reported the discontinuation of manufacture of nesiritide in 2018 [26].

Currently, we have an approved drug, a dual neprilysin/angiotensin receptor inhibitor, sacubitril/valsartan, for chronic HF, and neprilysin inhibition results in increased endogenous NPs, which is thought to play an important role in improving the prognosis in HF [27]. From the experiences with nesiritide and sacubitril/valsartan, we learned that long-term drug administration may be better at improving the prognosis, which is necessary for successful drug approval in HF.

It is difficult to develop oral drugs for peptides, such as insulin [28], and as such, they are administered intravenously or subcutaneously. Subcutaneous injection is affected by endocytosis, caused by subcutaneous degrading enzymes, such as neprilysin and NPR-C, which is related to pharmacokinetics, including transfer to the blood. Therefore, attempts have been made to design peptides that can withstand subcutaneous injection by suppressing degradation through changing the original structure. In addition to prolonging the half-life, novel NPs stimulate multiple receptors and can be expected to have various pharmacological actions. In this review, we focus on attempts to create novel NP system therapeutics designed for chronic administration.

## 2. Designing with Natriuretic Peptides

### 2.1. Dual GC-A and GC-B Activator: Cenderitide (CD-NP)

**Figure 2:**
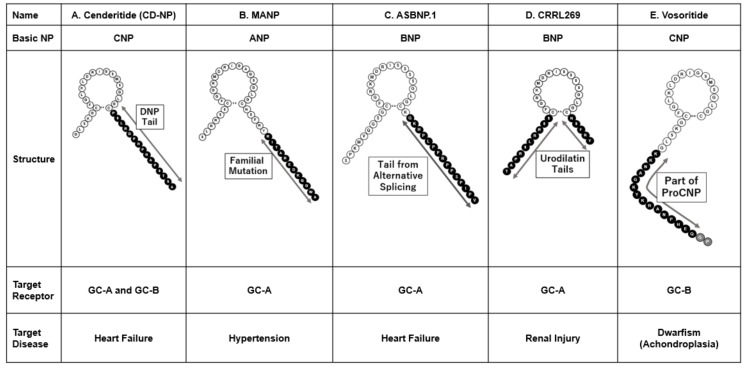
Designer NPs: Modified or combination of NPs. DNP, Dendroaspis natriuretic peptide; GC-A, guanylyl cyclase-A; GC-B, guanylyl cyclase-B.

To improve the prognosis in HF, therapeutic(s) may have inhibitory effects against myocardial remodeling and dysfunction. Cenderitide (CD-NP) was designed to have not only natriuresis, diuresis, and cardiovascular unloading effects via GC-A receptor activation, but also an anti-cardiac remodeling effect via GC-B receptor activation. Dendroaspis natriuretic peptide (DNP), presents in the eastern green mamba, had been identified as peptides with strong GC-A stimulation [29], but it was also confirmed that its 15 amino-acid C-terminal tail only can possess natriuretic and diuretic effects [30]. CD-NP, also called cenderitide, was designed as CNP added with the C-terminal tail of DNP (Figure 2A) [30].

It was confirmed in vitro that cenderitide stimulated cGMP production in both GC-A or GC-B over-expressing HEK cells, respectively [31], as well as significantly suppressed mRNA and protein expressions of type I collagen in cardiac fibroblasts compared to BNP and CNP [32]. In vivo experiments revealed that significantly increased natriuresis and diuresis were achieved by continuous intravenous infusion of cenderitide, which showed fewer hypotensive effects compared to BNP in normal canines [30]. The continuous intravenous administration of cenderitide for 4 weeks suppressed the myocardial fibrosis and decreased circulating aldosterone levels in 5/6 nephrectomy rats with myocardial fibrosis [31]. These results suggest that cenderitide has both anti-fibrotic/remodeling and natriuretic/diuretic actions via the dual activation of the GC-A and GC-B receptors.

Based on these results, a phase I clinical trial for cenderitide was conducted on 22 healthy subjects [33]. A 4 h continuous intravenous infusion of cenderitide resulted in increased circulating and urinary cGMP, natriuresis, and diuresis compared to placebo infusion, without a decrease in blood pressure (BP) or adverse events [33]. Further, in the phase II clinical trial of 18 patients with stable HF, continuous intravenous infusion for 4 h of cenderitide was performed following the same protocol as in phase I, and no significant difference in BP and heart rate was observed compared with the placebo, but a significant increase in diuretic and natriuretic effects was confirmed. An improvement in glomerular filtration rate (GFR) was also observed in patients with decreased renal function, suggesting cenderitide may have a renal protective effect for patients with renal failure [34].

The subcutaneous administration of cenderitide has been investigated as a method of chronic administration to reverse myocardial remodeling. Ichiki T et al. reported that subcutaneous continuous infusion of cenderitide for 10 days in HF canines induced by rapid right ventricular pacing resulted in a significant increase in cardiac output and eGFR compared to the untreated group and the group with the oral administration of an angiotensin converting enzyme (ACE) inhibitor, enalapril. In addition, no BP-lowering effect was observed [35]. A phase I study of cenderitide subcutaneous injection aimed at anti-myocardial remodeling was completed in 14 patients with end-stage HF supported with left ventricular assist devise [36]. A single subcutaneous dose of cenderitide (10 ug/kg) showed a tendency to lower BP compared to a placebo, but a significant NT-proBNP inhibitory effect and an increase in plasma cGMP over 4 h were observed, and no adverse events were reported [36]. Thus, cenderitide was produced based on the idea of stimulating dual NP receptors and confirmed the biological actions in preclinical and clinical studies, Now phase III trials for HF are warranted.

### 2.2. Long Acting ANP: MANP

The pharmaceutical formulation of human recombinant ANP, carperitide, has been used as a drug for acute decompensated HF in Japan. It has a significant natriuretic and diuretic effect, as well as a BP-lowering effect via GC-A activation [9], suggesting it can also be applied as a treatment for HT. In 2012, a case report of HT demonstrated the therapeutic subcutaneous administration of BNP (nesiritide) [37]. Three subcutaneous injections of nesiritide every 12 h were administered to patients with uncontrolled HT who already took two anti-hypertensive drugs, oral angiotensin receptor blocker and diuretic. The administration resulted in a marked decrease in BP [37]. It was further reported that there may be a relative deficiency of ANP and BNP in hypertensive patients in the general population in Olmsted County, MN [38]. These studies suggest that NP replacement therapy may be effective in HT. ANP, which has a strong diuretic effect, in addition to a vasodilatory effect, is a candidate as a novel drug for HT, but the issue of the subcutaneous injection of ANP is its short half-life due to its rapid metabolization in the subcutaneous area, resulting in a poor blood transfer rate [39]. A peptide that is resistant to degradation in the subcutaneous area should be required for long-term therapeutics.

A peptide was discovered in 2008 from patients with familial atrial fibrillation with a mutation in the ANP gene. The gene mutation added 12 amino acids to the C-terminus of ANP, called mutant-ANP (MANP) (Figure 2B) [40]. McKie et al. reported that MANP stimulated intracellular cGMP activity in human cardiac fibroblasts compared to ANP [41]. Continuous intravenous infusion of MANP in normal canines revealed a sustained BP decrease and natriuretic and diuretic effects with RAAS inhibitory actions compared to an equimolar dose of ANP [41]. Furthermore, MANP showed dose-dependent cGMP-stimulating actions in vitro in GC-A overexpressing HEK cells, human aortic smooth muscle cells, and human aortic endothelial cells [42,43]. In addition, Dickey et al. confirmed that MANP has similar GC-A receptor or NPR-C binding activity to ANP; however, MANP was more resistant to neprilysin-induced proteolytic degradation, which may be the main mechanism of its longer half-life than ANP [44]. Preclinical in vivo studies revealed that continuous intravenous infusion of MANP led to an increase in and the prolongation of diuretic, natriuretic, and antihypertensive effects, together with aldosterone suppression in normal canines [41] and canines with hypertensive HF caused by angiotensin II intravenous infusion [45]. Further, MANP was investigated for more applicable administrations than intravenous infusion for the clinical setting in chronic diseases, including HT. Once-daily subcutaneous administration of MANP for 7 days for salt-sensitive hypertensive rats resulted in a dose-dependent BP-lowering and diuretic effect and aldosterone inhibition [42]. Dzhoyashivili et al. also reported a study on the co-intravenous infusion of MANP and furosemide in spontaneously hypertensive rats [43]. MANP augmented BP-lowering effects by furosemide, in addition to suppressing increased circulating aldosterone levels by furosemide infusion. These preclinical studies suggest that MANP has prolonged BP-lowering and aldosterone-suppressive effects via GC-A activation.

Based on these pre-clinical results, the first in-human study of MANP for essential hypertensive patients was reported by Chen et al. in 2022 [46]. The study was designed as an open-label, sequential single-ascending-dose study to determine the safety, tolerability, neurohumoral, renal, and BP-lowering properties of MANP. After ceasing anti-hypertensive medications for 2 weeks, patients were given a single shot of MANP, administered subcutaneously; then, data were observed and collected for vital signs, blood, and urine to measure neurohumoral factors up to 24 h. MANP administration activated circulating and urinary cGMP, induced natriuresis, reduced aldosterone, and decreased BP at or below the maximal tolerated dose, defined as a systolic BP reduction of ≥30 mmHg, without any adverse events [46].

MANP’s action in HT sounds promising, and a phase II clinical trial for hypertensive patients will be expected. If MANP succeeds in HT, it may be applicable for therapeutics in hypertensive HF.

### 2.3. BNP Based Peptides

#### 2.3.1. ASBNP.1 (ANX-042)

In the clinical setting, NP injection/infusion sometimes causes excessive hypotension which may cause further organ failure in HF. To avoid the hypotensive effects, NP without BP-lowering effect is required. The peptide forms from alternative RNA splicing may have a unique diagnostic or therapeutic opportunity. Pan et al. identified an alternative spliced transcript form of BNP which presented in the failing human heart [47]. The 60 amino acid alternative spliced BNP (ASBNP) lacks cGMP synthetization in human umbilical endothelial cells, and vasorelaxation ex vivo rabbit carotid arterial rings, suggesting ASBNP may lose the dose-dependent BP-lowering effects of BNP. After the structural considerations, Pan et al. shortened the C-terminal tail of the ASBNP into 42 amino acid ASBNP.1 (Figure 2C). Similar to ASBNP, ASBNP.1 did not have BP-lowering effects but did increase GFR and urinary and diuretic effects, and suppressed plasma renin and angiotensin II levels [47]. These results suggest that ASBNP.1 may have renal enhancing effects without vasodilatation, and ASBNP.1 is currently in phase I clinical trial [48].

#### 2.3.2. CRRL269

Cenderitide and MANP are peptides designed from CNP and ANP, respectively, but there is a designer peptide originally from BNP. This peptide, called CRRL269, has only the ring structure of BNP and the C-terminal and N-terminal tails of urodilatin, which is one of the precursors of ANP, as well as an additional four amino acids on the N-terminal end (Figure 2D) [49]. CRRL269 was designed with the expectation of it having a more diuretic effect from urodilatin or ANP, in addition to the action of BNP, through this modification.

CRRL269 demonstrated a cGMP production equivalent to BNP and urodilatin in GC-A receptor overexpressed HEK cells, and its enzymatic degradation by neprilysin was suppressed compared to urodilatin [49]. In addition, continuous intravenous injection of CRRL269 in normal canines enhanced and prolonged the natriuretic and diuretic effect compared to urodilatin without hypotensive effects [49]. Because CRRL269 may not have an antihypertensive effect and but may prolong action on the kidney, Yang et al. created a canine acute kidney injury model to test its therapeutic application to renal injury [50]. When CRRL269 or a placebo was administered for 2 h to canines with acute renal injury by clamping the abdominal aorta on the renal artery bifurcation for 1 h, enhancement in circulating cGMP levels, improvement in natriuresis, diuresis, GFR, and angiotensin II levels after renal injury, and reduced cardiac filling pressure were observed without hypotensive effects compared to the placebo. Apoptosis in renal tissues after kidney injury was also suppressed, together with reduced proapoptotic gene expressions compared to the placebo [50]. These results suggest that CRRL269 serves as a novel reno-cardiac protective agent for acute kidney injury. Although this peptide has not yet reached clinical trials, it is expected to be clinically applied as a drug for acute renal disease.

### 2.4. Long-Acting CNP

#### 2.4.1. Vosoritide

CNP plays a key role in bone growth, as demonstrated in GC-B receptor knockout mice, which showed bone dysplasia and were fetal-lethal, and even CNP knockout mice, half of which are fetal-lethal and the rest show severe dwarfism due to endochondral ossification disorder [5,6]. Therefore, CNP is thought to be crucial in bone growth before epiphyseal formation closure [5,6]. Therapeutic application of CNP for dwarfism in children can be considered, but CNP is not suitable for subcutaneous injection or chronic use, as it is greatly affected by enzymatic degradation via neprilysin and it has a short half-life.

The CNP precursor proCNP is converted to CNP1-53, consisting of 53 amino acids, by its converting enzyme furin, and it is then further converted by an unknown enzyme into active CNP, consisting of 22 amino acids [5,6]. Vosoritide (also called BMN111), a 39 amino-acid peptide, is an analog of human CNP (CNP1-22) that corresponds to amino acid sequences of 17–53 in human CNP1-53, with an additional two amino acids in the N-terminal end (Figure 2E) [51].

Achondroplasia, a common genetic disease of dwarfism, is characterized by rhizomatic shortening of the limbs and macrocephaly. Achondroplasia is caused by a gain-of-function mutation in the fibroblast growth factor receptor 3 (FGFR3) gene [52], and CNP can inhibit FGFR3 signaling by suppressing the MAPK pathway [51]. Treatment with vosoritide resulted in a significant recovery of bone growth in the FGFR3 mouse model, which showed disproportionate dwarfism [51]. Wendt et al. confirmed that vosoritide showed significant resistance to degradation by not only CNP1-22 but also other CNP-based analogs in vitro [53]. Both intravenous and subcutaneous injections of vosoritide resulted in a longer serum half-life compared to CNP1-22 in mice and rats. Further, they confirmed that daily subcutaneous injections of vosoritide in juvenile cynomolgus monkeys led to accelerated bone growth and good tolerance in hemodynamics [53].

In 2019, Savarirayan et al. reported a successful multicenter phase II clinical trial as a dose-finding study, conducted for 5–14-year-old children with achondroplasia [54]. Once-daily subcutaneous administration of vosoritide resulted in a sustained increase in the annualized growth velocity, with generally mild adverse events. A successful randomized, double-blind, phase 3, placebo-controlled study for 5–14-year-old children with achondroplasia was then reported in 2020 [55]. The patients received a 15 ug/kg daily subcutaneous administration of vosoritide for 52 weeks, wherein no deaths or serious adverse events were observed and treated the group showed significant growth velocity [55]. According to these successful phase II and III clinical trials, vosoritide was approved as a therapeutic in patients with achondroplasia in the European Union in August 2021 and then in the US in November 2021.

#### 2.4.2. TransCon CNP

Further long-acting CNP synthesis was performed using a technique called TransCon, which prolongs the conversion of the precursor peptide to the active form and slowly releases the active peptide. TransCon CNP is a linker of two amino acids and branched polyethylene glycol (PEG) on CNP1-38, which has an additional 18 amino acids on the N-terminus of CNP [56].

Breinholt et al. reported that TransCon CNP was resistant to enzymatic degradation by neprilysin and had a long half-life compared to CNP1-38 or vosoritide [56]. Daily subcutaneous administration of TransCon CNP did not show any cardiovascular effects in mice and cynomolgus monkeys, whereas CNP1-38 or vosoritide resulted in a decrease in BP and an increase in heart rate. Further, the subcutaneous weekly administration of TransCon CNP for 26 weeks promoted bone growth in monkeys [56]. TransCon Technology allowed the weekly administration of TransCon CNP due to the slow release of active CNP, and the preclinical study confirms the safety and efficacy of TransCon CNP therapeutics for bone growth.

## 3. Designing Natriuretic Peptides with Peptides from Other Systems

### 3.1. NPA7

The combination of two NPs results in the additional effect of prolonging and enhancing the action or stimulation of two types of receptors, such as cenderitide. Therefore, the synthesis of peptides targeting not only the NP system but receptors in other systems is possible.

**Figure 3:**
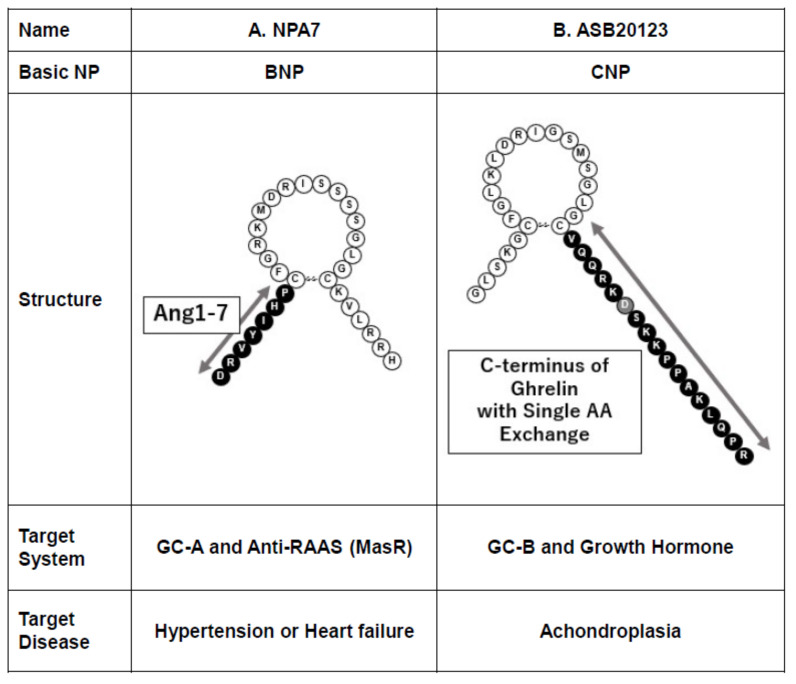
Designer NPs: Combination of NP and peptide from other systems. AA, amino acid; Ang1–7, angiotensin1–7, GC-A, guanylyl cyclase-A, GC-B, guanylyl cyclase-B; MasR, Mas receptor; RAAS, renin-angiotensin-aldosterone system.

RAAS is an important therapeutic target that is an exacerbating hormone system, which affects, for example, heart disease, HT, atherosclerosis, or organ fibrosis. Ang1–7, a degradation product of angiotensin II, is thought to be an antagonist of RAAS and is also known to have a cardiovascular protective effect via the Mas receptor [57]. However, it is too small a peptide, consisting of only seven amino acids, so it was difficult to develop into a therapeutic drug due to its too short half-life. NPA7 is a peptide that replaces the N-terminal tail of BNP with Ang1–7, which was expected to have a longer half-life than Ang1–7 and an organ-protective effect from both the Mas receptor and GC-A receptor stimulation (Figure 3A) [58].

NPA7 strongly stimulated GC-A receptor overexpressing HEK cells with the activation of intracellular cGMP, but it also bound to HEK cells with a strong expression of the Mas receptor by generating its second messenger, cAMP. When Ang1–7 alone was continuously infused into normal canines, no cardiovascular effect or diuretic effect was observed, but NPA7 significantly enhanced BP-lowering, natriuretic, and cardiac-unloading effects compared to BNP [58]. According to these results, NPA7 is not only “super BNP,” but it also can be expected to have a further cardiovascular-protective effect via the Mas receptor. Further preclinical studies are warranted.

### 3.2. ASB20123

Although CNP is a bone-growth-promoting hormone and has been described as having clinical applicability for dwarfism in children, growth hormones are also an important hormone for bone growth before epiphyseal plate closure. Ghrelin is produced mainly in the gastrointestinal mucosa and is known as a feeding-promoting peptide, but ghrelin is also secreted in the hypothalamus. Ghrelin directly stimulates the secretion of growth hormone in the pituitary gland, and it stimulates the secretion of a growth hormone-stimulating hormone in the hypothalamus, and then stimulates the release of growth hormone in the pituitary grand [59].

ASB20123 is a CNP-based peptide developed in Japan (Figure 3B) [60]. The C-terminus of ghrelin with single amino acid exchange was added to the C-terminal end of the ring of CNP, which resulted in the growth hormone secretagogue action of ghrelin in addition to the CNP function. It is also intended to be applied in the treatment of dwarfism, much like vosoritide (Figure 3B) [60].

ASB20123 stimulated intracellular cGMP production in Chinese hamster ovary (CHO) cells expressing the GC-B receptor in the same manner as CNP to produce cGMP. The half-life of a single intravenous injection of ASB20123 was 6.7 times as long as CNP, and a single subcutaneous injection of ASB20123 also showed an increase in plasma cGMP over 3 h. The effect of bone growth has been confirmed in young mice and rats [60], and it has also been confirmed that there is no bone toxicity [61]. This peptide is also expected to be clinically applied to dwarfism, and early clinical trials are warranted.

## 4. Oral Drugs as Modulators for Endogenous Natriuretic Peptides

### 4.1. Difficulty in Developing Oral Drugs for Peptides

Ideally, the development of an oral drug has been desired as a drug delivery method for the long-term administration of NP. In fact, studies on oral BNP drugs have also been published, confirming an increase in plasma cGMP concentration and an antihypertensive effect following oral administration to normal canines [62]. However, the cost of producing oral drugs then was significantly high, and the absorption efficiency of the trans-digestive tract was also poor, so subsequent development did not proceed.

Insulin is also a famous therapeutic peptide for diabetes mellitus; it is widely used for subcutaneous injection rather than NPs, as it has been extremely difficult to develop an oral form of insulin. Each gastrointestinal tract has different absorption patterns and degrading enzymes from the stomach, small intestine, and large intestine, and there are also large individual differences. Novo Nordisk’s I338, the most famous oral insulin drug, also progressed to Phase II with an incredibly low absorption efficiency of 1.5–2% while obtaining a good hypoglycemic effect [63]. Unfortunately, the development was discontinued because it was not cost-effective, because a large amount of peptide synthesis was required to obtain the biological effect [28]. Although several other oral insulin drugs are under development, clinical application is unlikely at this time unless breakthrough technologies are developed to prevent peptide degradation in gastrointestinal absorption.

### 4.2. Sacubitril/Valsartan

The first approved oral drug in the NP system is a combined angiotensin receptor and neprilysin inhibitor, sacubitril/valsartan. Substrates for neprilysin include NPs (kinetic affinity CNP = ANP > BNP) [5], angiotensin, adrenomedullin, substance P, etc. The inhibition of neprilysin prolongs the half-life of endogenous natriuretic peptides together with angiotensin II, so the co-inhibition of RAAS was required for therapeutics in HF. The PARADIGM-HF, phase III, randomized, double-blind trial, which compared to an ACE inhibitor, enalapril in HF patients with reduced ejection fraction, resulted in a reduced risk of death and of rehospitalization for HF, together with reduced circulating levels of NT-proBNP in 2014 [27]. The drug was approved for medical use for HF in more than 110 countries in 2022 and for HT in Japan, Russia, and China. Anti-cardiac remodeling effects, together with enhancing natriuretic peptides, were reported by Januzzi et al. in 2019 [64]. This phase IV multicenter clinical trial, the PROVE-HF trial in the US, was designed to determine the NT-proBNP-lowering effects of sacubitril/valsartan together with the change in cardiac structure and function in HF patients with reduced EF. After 12 months of oral treatment with sacubitril/valsartan, there was a decrease in NT-proBNP, which correlated with improved left ventricular systolic function and end-diastolic volume [64]. The sub-analysis of PROVE-HF in HF patients with reduced EF revealed a significant increase in circulating ANP levels, which was associated with an improvement in cardiac remodeling [65]; however, Solomon et al. reported that sacubitril/valsartan did not improve the risk of death and rehospitalization in patients with preserved EF from the phase III PARAGON-HF trial [66]. The sub-analysis from both PARADIGM and PARAGON-HF trial revealed that the improvement of prognosis by the treatment with sacubitril/valsartan appeared in the patient below the normal limit of EF [67]. Currently, the use of endogenous NP therapeutic with sacubitril/valsartan is a class I recommendation for HF with reduced EF as a replacement for an ACE inhibitor or angiotensin receptor blocker [68]. The important question of why sacubitril/valsartan was not effective in HF with preserved EF should be clarified in future studies in order to understand the pathophysiology of NP system in HF with preserved EF.

### 4.3. Small Molecules Targeting GC-A Receptor

#### 4.3.1. GC-A Activator

A small molecule is one option for creating an oral drug for the NP system. Iwaki et al. reported GC-A activator for the first time in 2017 [69]. After modification of triazine derivatives by dimerization, two molecules showed enhanced cGMP levels in GC-A overexpressing CHO cells and these continuous infusions increased diuresis comparable to 1 ug/kg/min ANP in rats. Since these molecules were very high molecular weight and not suitable for drug development, Iwaki et al. performed high-throughput screening from the compound library in the Daiichi Sankyo as GC-A activator using GC-A overexpressing CHO cells [70]. Two molecules were discovered, and the scaffold hopping of thienopyridine to quinazoline and additional optimization of the substituent on the 6-position of the benzene ring of quinazoline increased the GC-A agonistic activity 350 times compared to that of the original compound. Because the modified compound had low GC-A activity in rats, Iwaki et al. reported on the further modification [71]. The quinazoline and pyrido[2,3-d]pyrimidine derivatives showed potency as a GC-A activator together with the equivalent in vivo urinary effects to 1 ug/kg/min ANP in rats. The small molecule as a GC-A activator would be a potential therapeutic for HF or HT, and further preclinical and clinical studies are warranted.

#### 4.3.2. GC-A Positive Allosteric Modulator, MCUF-651

Sangaralingham et al. reported the novel discovery of a GC-A receptor modulator in 2022 [72]. In a cell-based high-throughput screening of the NIH Molecular Libraries Small Molecule Repository (370,620 compounds), GC-A positive allosteric modulator (PAM) scaffolds were identified. The medicinal chemistry structure–activity relationship efforts of the lead scaffold actually found a GC-A PAM, MCUF-651. Although MCUF-651 could not activate cGMP production in GC-A or GC-B overexpressed HEK cells without ANP or CNP, respectively, it further activated cGMP production in GC-A HEK cells co-treated with ANP. MCUF-651 could enhance the binding affinity of ANP and BNP in GC-A, but not CNP in GC-B receptors. The MCUF-651 dose-dependent augmentation of cGMP production by ANP was observed in human renal proximal tubular cells, human visceral adipocytes, and human cardiomyocytes. MCUF-651 was orally bioavailable in mice in vivo, and there were enhanced endogenous ANP and BNP ex vivo actions in the plasma of normal subjects and in patients with HT and HF [72]. Although the direct activator in GC-A or GC-B cells was not found, the GC-A PAM, MCUF-651, has great potential as a novel oral therapeutic.

### 4.4. How to Use Novel Drugs of NP Systems, Properly?

After learning about these great attempts at creating novel GC-A or GC-B stimulators, modulators, or endogenous NPs enhancer, there is the question of which is the better option for therapeutics. In fact, one of the endogenous NPs enhancers, sacubitril/valsartan, was already approved as an oral drug, and it may have better medication compliance with easier application and less pain than subcutaneous administration for patients. The key points would be whether diseased patients have enough endogenous NPs.

Machret et al. reported the relative deficiency of ANP and BNP in human HT in 2012 [73]. Plasma levels of ANP and BNP molecular forms were determined in the 2082 general population in Olmsted County, MN. Compared to normotensive patients, prehypertensive patients had lower BNP, NT-proBNP, and NT-proANP levels. In the same population, Buglioni et al. reported that higher levels of aldosterone had associations with HT, obesity, chronic kidney disease, metabolic syndrome, atrial fibrillation, and concentric left ventricular hypertrophy, together with lower NP levels [74]. Further, Asferg et al. also reported that 63 obese, hypertensive men with Body mass index (BMI) ≥ 30 and BP ≥ 130/80 mmHg showed lower serum levels of mid-regional proANP levels, whereas there was no significant change in renin or angiotensin II levels compared to 27 lean, normotensive men [75].

Interestingly, a relative deficiency in NPs was also reported in HF, although circulating NPs were sensitive biomarkers for diagnostics in HF. Reginauld et al. reported that 26% of 112 acute decompensate HF patients had normal levels of ANP and relatively low cGMP levels. The HF group with normal ANP value had higher BMI and EF than others [76]. Bachmann et al. also reported that among 13,613 patients, extremely low BNP levels were observed in 4.9%, 14.0%, and 16.3% of patients with hospitalized HF, abnormal cardiac structure/function, or abnormal hemodynamics, respectively. The strongest predictor of low BNP levels was obesity, and no BNP gene-coding variations were observed in these patients [77]. These results suggest that a relative deficiency of NP may exist in some populations in HT, obesity, metabolic syndrome, and HF, and these patients may be a group of “non-responders” to therapeutic NP receptor modulators or endogenous NP enhancers. Therefore, for the relative NP deficiency group, direct NP stimulators, including designer NP, would be suitable.

## 5. Future Prospects for Drug Discovery of Natriuretic Peptides

### 5.1. Safety Verification

Designing NPs means creating a substance that naturally does not exist in the human body. There is a concern that the administration of “designer peptides” may activate the immune system as a defense mechanism and antibodies against new peptides will then be produced, resulting in the occurrence of adverse events and/or diminishing drug efficacy. In addition, structural changes themselves may diminish drug efficacy, regardless of the immune response. Because it is undeniable that any adverse events may occur in humans even if they are not toxic in animal experiments, safety verification must be performed carefully at every stage of the clinical trial.

### 5.2. Barriers to Patent Validity and the Enormous Costs for Clinical Trials

Exploring pathophysiology and then discovering and developing therapeutic agents is a brilliant goal of medical research, but achieving it is exceedingly difficult. For drug discovery, we obtain a patent by accumulating basic experimental data from substance discovery and design; then, we must prove efficacy and safety from animal experiments and finally proceed to review for drug approval after obtaining phases I to III of clinical trials. First, preclinical trials require a considerable cost and time, where each clinical trial costs a huge amount of money. DiMost et al. reported that capitalized costs (including facility, equipment, and maintenance, as well as human costs) were USD 1 billion for preclinical studies and about USD 100 billion from phase I to final approval. Unfortunately, only 11.8% are eventually approved [78]. It will be an endless battle between the time to work as a researcher and utilizing laboratory resources, high costs, and the end of patency.

It is usually difficult for a developed laboratory to take charge of everything up to phase III, and it is necessary to outsource to a pharmaceutical company at some point. On the other hand, pharmaceutical companies must also recover the enormous costs leading up to phase III and drug approval and make a profit during the period when patent-valid drugs can be exclusively sold. Because there is a limit to profit recovery when selling patents from a single country, it is also necessary to obtain international patents that are expected to be sold worldwide.

It seems that many new drugs have been abandoned due to cost or time constraints, even if the efficacy and safety are confirmed up to phase I or phase II. If a system supports laboratories and provides a foothold for pharmaceutical companies, it seems pharmaceutical development will progress dramatically.

## 6. Conclusions

Although there are various difficulties, designing new NPs, including both direct stimulators of NP receptors and endogenous NP system modulators, has been confirmed for clinical application as an effective therapeutic in some disease states. Attempts to create better drugs are still ongoing, and further advances in drug development systems are expected, as are better prognoses in human beings.

## References

[1] De Bold A.J. (1979). Heart atria granularity effects of changes in water-electrolyte balance. Proc. Soc. Exp. Biol Med..

[2] de Bold A.J. (1982). Atrial natriuretic factor of the rat heart. Studies on isolation and properties. Proc. Soc. Exp. Biol. Medicine. Soc. Exp. Biol. Med..

[3] Kangawa K., Matsuo H. (1984). Purification and complete amino acid sequence of alpha-human atrial natriuretic polypeptide (alpha-hANP). Biochem. Biophys. Res. Commun..

[4] Sudoh T., Minamino N., Kangawa K., Matsuo H. (1990). C-type natriuretic peptide (CNP): A new member of natriuretic peptide family identified in porcine brain. Biochem. Biophys. Res. Commun.

[5] Potter L.R. (2011). Natriuretic peptide metabolism, clearance and degradation. FEBS J..

[6] Yasoda A., Nakao K. (2010). Translational research of C-type natriuretic peptide (CNP) into skeletal dysplasias. Endocr. J..

[7] Saito Y. (2010). Roles of atrial natriuretic peptide and its therapeutic use. J. Cardiol..

[8] Ichiki T., Huntley B.K., Burnett J.C. (2013). BNP molecular forms and processing by the cardiac serine protease corin. Adv. Clin. Chem..

[9] Ichiki T., Burnett J.C. (2017). Atrial Natriuretic Peptide- Old But New Therapeutic in Cardiovascular Diseases. Circ. J..

[10] Bordicchia M., Liu D., Amri E.Z., Ailhaud G., Dessi-Fulgheri P., Zhang C., Takahashi N., Sarzani R., Collins S. (2012). Cardiac natriuretic peptides act via p38 MAPK to induce the brown fat thermogenic program in mouse and human adipocytes. J. Clin. Investig..

[11] Miyashita K., Itoh H., Tsujimoto H., Tamura N., Fukunaga Y., Sone M., Yamahara K., Taura D., Inuzuka M., Sonoyama T. (2009). Natriuretic peptides/cGMP/cGMP-dependent protein kinase cascades promote muscle mitochondrial biogenesis and prevent obesity. Diabetes.

[12] Coue M., Barquissau V., Morigny P., Louche K., Lefort C., Mairal A., Carpene C., Viguerie N., Arner P., Langin D. (2018). Natriuretic peptides promote glucose uptake in a cGMP-dependent manner in human adipocytes. Sci. Rep..

[13] Ferraino K.E., Cora N., Pollard C.M., Sizova A., Maning J., Lymperopoulos A. (2021). Adrenal angiotensin II type 1 receptor biased signaling: The case for “biased” inverse agonism for effective aldosterone suppression. Cell Signal..

[14] Guitart-Mampel M., Urquiza P., Borges J.I., Lymperopoulos A., Solesio M.E.E. (2021). Impact of Aldosterone on the Failing Myocardium: Insights from Mitochondria and Adrenergic Receptors Signaling and Function. Cells.

[15] Kuwahara K. (2021). The natriuretic peptide system in heart failure: Diagnostic and therapeutic implications. Pharmacol. Ther..

[16] Miura S., Nakayama A., Tomita S., Matsuo Y., Suematsu Y., Saku K. (2015). Comparison of aldosterone synthesis in adrenal cells, effect of various AT1 receptor blockers with or without atrial natriuretic peptide. Clin. Exp. Hypertens.

[17] Moyes A.J., Hobbs A.J. (2019). C-type Natriuretic Peptide: A Multifaceted Paracrine Regulator in the Heart and Vasculature. Int. J. Mol. Sci..

[18] Kuhn M. (2016). Molecular Physiology of Membrane Guanylyl Cyclase Receptors. Physiol Rev..

[19] Friebe A., Sandner P., Schmidtko A. (2020). cGMP: A unique 2nd messenger molecule—Recent developments in cGMP research and development. Naunyn Schmiedebergs Arch. Pharm..

[20] Publication Committee for the VMAC Investigators (2002). Intravenous nesiritide vs nitroglycerin for treatment of decompensated congestive heart failure: A randomized controlled trial. JAMA.

[21] Hayashi M., Tsutamoto T., Wada A., Maeda K., Mabuchi N., Tsutsui T., Horie H., Ohnishi M., Kinoshita M. (2001). Intravenous atrial natriuretic peptide prevents left ventricular remodeling in patients with first anterior acute myocardial infarction. J. Am. Coll. Cardiol..

[22] Mohammed S.F., Korinek J., Chen H.H., Burnett J.C., Redfield M.M. (2008). Nesiritide in acute decompensated heart failure: Current status and future perspectives. Rev. Cardiovasc Med..

[23] Nomura F., Kurobe N., Mori Y., Hikita A., Kawai M., Suwa M., Okutani Y. (2008). Multicenter prospective investigation on efficacy and safety of carperitide as a first-line drug for acute heart failure syndrome with preserved blood pressure: COMPASS: Carperitide Effects Observed Through Monitoring Dyspnea in Acute Decompensated Heart Failure Study. Circ. J..

[24] Hata N., Seino Y., Tsutamoto T., Hiramitsu S., Kaneko N., Yoshikawa T., Yokoyama H., Tanaka K., Mizuno K., Nejima J. (2008). Effects of carperitide on the long-term prognosis of patients with acute decompensated chronic heart failure: The PROTECT multicenter randomized controlled study. Circ. J..

[25] O’Connor C.M., Starling R.C., Hernandez A.F., Armstrong P.W., Dickstein K., Hasselblad V., Heizer G.M., Komajda M., Massie B.M., McMurray J.J.V. (2011). Effect of nesiritide in patients with acute decompensated heart failure. N. Engl. J. Med..

[26] Kittleson M.M. (2018). Nesiritide and Me. Circ. Heart Fail..

[27] McMurray J.J., Packer M., Desai A.S., Gong J., Lefkowitz M.P., Rizkala A.R., Rouleau J.L., Shi V.C., Solomon S.D., Swedberg K. (2014). Angiotensin-neprilysin inhibition versus enalapril in heart failure. N. Engl. J. Med..

[28] Drucker D.J. (2020). Advances in oral peptide therapeutics. Nat. Rev. Drug Discov..

[29] Schweitz H., Vigne P., Moinier D., Frelin C., Lazdunski M. (1992). A new member of the natriuretic peptide family is present in the venom of the green mamba (*Dendroaspis angusticeps*). J. Biol. Chem..

[30] Lisy O., Huntley B.K., McCormick D.J., Kurlansky P.A., Burnett J.C. (2008). Design, synthesis, and actions of a novel chimeric natriuretic peptide: CD-NP. J. Am. Coll. Cardiol..

[31] Martin F.L., Sangaralingham S.J., Huntley B.K., McKie P.M., Ichiki T., Chen H.H., Korinek J., Harders G.E., Burnett J.C. (2012). CD-NP: A novel engineered dual guanylyl cyclase activator with anti-fibrotic actions in the heart. PLoS ONE.

[32] Ichiki T., Schirger J.A., Huntley B.K., Brozovich F.V., Maleszewski J.J., Sandberg S.M., Sangaralingham S.J., Park S.J., Burnett J.C. (2014). Cardiac fibrosis in end-stage human heart failure and the cardiac natriuretic peptide guanylyl cyclase system: Regulation and therapeutic implications. J. Mol. Cell. Cardiol..

[33] Lee C.Y., Chen H.H., Lisy O., Swan S., Cannon C., Lieu H.D., Burnett J.C. (2009). Pharmacodynamics of a novel designer natriuretic peptide, CD-NP, in a first-in-human clinical trial in healthy subjects. J. Clin. Pharm..

[34] Kawakami R., Lee C.Y.W., Scott C., Bailey K.R., Schirger J.A., Chen H.H., Benike S.L., Cannone V., Martin F.L., Sangaralingham S.J. (2018). A Human Study to Evaluate Safety, Tolerability, and Cyclic GMP Activating Properties of Cenderitide in Subjects With Stable Chronic Heart Failure. Clin. Pharm..

[35] Ichiki T., Schirger J.A., Wanek J.R., Heublein D.M., Scott C.G., Sangaralingham S.J., Chen H.H., Burnett J.C. (2018). Cardiorenal protection by subcutaneous cenderitide in experimental heart failure: A novel and safe therapeutic for humans with LVAD support. Eur. Heart J..

[36] Ichiki T., Schirger J., Wanek J.R., Scott C., Sangaralingham J., Chen H.H., Burnett J. (2020). Cenderitide: A Novel Therapeutic to Increase Endogenous Cardiac Natriuretic Peptides in Heart Failure. J. Am. Coll. Cardiol..

[37] Cataliotti A., Costello-Boerrigter L.C., Chen H.H., Textor S.C., Burnett J.C. (2012). Sustained blood pressure-lowering actions of subcutaneous B-type natriuretic peptide (nesiritide) in a patient with uncontrolled hypertension. Mayo Clin. Proc..

[38] Macheret F., Heublein D., Costello-Boerrigter L.C., Boerrigter G., McKie P., Bellavia D., Mangiafico S., Ikeda Y., Bailey K., Scott C.G. (2012). Human hypertension is characterized by a lack of activation of the antihypertensive cardiac hormones ANP and BNP. J. Am. Coll. Cardiol..

[39] Crozier I.G., Nicholls M.G., Ikram H., Espiner E.A., Yandle T.G. (1987). Plasma immunoreactive atrial natriuretic peptide levels after subcutaneous alpha-hANP injection in normal humans. J. Cardiovasc. Pharm..

[40] Hodgson-Zingman D.M., Karst M.L., Zingman L.V., Heublein D.M., Darbar D., Herron K.J., Ballew J.D., de Andrade M., Burnett J.C., Olson T.M. (2008). Atrial natriuretic peptide frameshift mutation in familial atrial fibrillation. N. Engl. J. Med..

[41] McKie P.M., Cataliotti A., Huntley B.K., Martin F.L., Olson T.M., Burnett J.C. (2009). A human atrial natriuretic peptide gene mutation reveals a novel peptide with enhanced blood pressure-lowering, renal-enhancing, and aldosterone-suppressing actions. J. Am. Coll. Cardiol..

[42] Chen Y., Schaefer J.J., Iyer S.R., Harders G.E., Pan S., Sangaralingham S.J., Chen H.H., Redfield M.M., Burnett J.C. (2020). Long-term blood pressure lowering and cGMP-activating actions of the novel ANP analog MANP. Am. J. Physiol. Regul. Integr. Comp. Physiol..

[43] Dzhoyashvili N.A., Iyer S.R., Chen H.H., Burnett J.C. (2022). MANP (M-Atrial Natriuretic Peptide) Reduces Blood Pressure and Furosemide-Induced Increase in Aldosterone in Hypertension. Hypertension.

[44] Dickey D.M., Yoder A.R., Potter L.R. (2009). A familial mutation renders atrial natriuretic Peptide resistant to proteolytic degradation. J. Biol. Chem..

[45] McKie P.M., Cataliotti A., Boerrigter G., Chen H.H., Sangaralingham S.J., Martin F.L., Ichiki T., Burnett J.C. (2010). A novel atrial natriuretic peptide based therapeutic in experimental angiotensin II mediated acute hypertension. Hypertension.

[46] Chen H.H., Wan S.H., Iyer S.R., Cannone V., Sangaralingham S.J., Nuetel J., Burnett J.C. (2021). First-in-Human Study of MANP: A Novel ANP (Atrial Natriuretic Peptide) Analog in Human Hypertension. Hypertension.

[47] Pan S., Chen H.H., Dickey D.M., Boerrigter G., Lee C., Kleppe L.S., Hall J.L., Lerman A., Redfield M.M., Potter L.R. (2009). Biodesign of a renal-protective peptide based on alternative splicing of B-type natriuretic peptide. Proc. Natl. Acad. Sci. USA.

[48] Meems L.M.G., Burnett J.C. (2016). Innovative Therapeutics: Designer Natriuretic Peptides. JACC Basic Transl. Sci..

[49] Chen Y., Harty G.J., Huntley B.K., Iyer S.R., Heublein D.M., Harders G.E., Meems L., Pan S., Sangaralingham S.J., Ichiki T. (2018). CRRL269: A novel designer and renal-enhancing pGC-A peptide activator. Am. J. Physiol. Regul. Integr. Comp. Physiol..

[50] Chen Y., Harty G.J., Zheng Y., Iyer S.R., Sugihara S., Sangaralingham S.J., Ichiki T., Grande J.P., Lee H.C., Wang X. (2019). Crrl269. Circ. Res..

[51] Lorget F., Kaci N., Peng J., Benoist-Lasselin C., Mugniery E., Oppeneer T., Wendt D.J., Bell S.M., Bullens S., Bunting S. (2012). Evaluation of the therapeutic potential of a CNP analog in a Fgfr3 mouse model recapitulating achondroplasia. Am. J. Hum. Genet..

[52] Rousseau F., Bonaventure J., Legeai-Mallet L., Pelet A., Rozet J.M., Maroteaux P., Le Merrer M., Munnich A. (1994). Mutations in the gene encoding fibroblast growth factor receptor-3 in achondroplasia. Nature.

[53] Wendt D.J., Dvorak-Ewell M., Bullens S., Lorget F., Bell S.M., Peng J., Castillo S., Aoyagi-Scharber M., O’Neill C.A., Krejci P. (2015). Neutral Endopeptidase-Resistant C-Type Natriuretic Peptide Variant Represents a New Therapeutic Approach for Treatment of Fibroblast Growth Factor Receptor 3-Related Dwarfism. J. Pharm. Exp. Ther..

[54] Savarirayan R., Irving M., Bacino C.A., Bostwick B., Charrow J., Cormier-Daire V., Le Quan Sang K.H., Dickson P., Harmatz P., Phillips J. (2019). C-Type Natriuretic Peptide Analogue Therapy in Children with Achondroplasia. N. Engl. J. Med..

[55] Savarirayan R., Tofts L., Irving M., Wilcox W., Bacino C.A., Hoover-Fong J., Ullot Font R., Harmatz P., Rutsch F., Bober M.B. (2020). Once-daily, subcutaneous vosoritide therapy in children with achondroplasia: A randomised, double-blind, phase 3, placebo-controlled, multicentre trial. Lancet.

[56] Breinholt V.M., Rasmussen C.E., Mygind P.H., Kjelgaard-Hansen M., Faltinger F., Bernhard A., Zettler J., Hersel U. (2019). TransCon CNP, a Sustained-Release C-Type Natriuretic Peptide Prodrug, a Potentially Safe and Efficacious New Therapeutic Modality for the Treatment of Comorbidities Associated with Fibroblast Growth Factor Receptor 3-Related Skeletal Dysplasias. J. Pharm. Exp. Ther..

[57] Patel V.B., Zhong J.C., Grant M.B., Oudit G.Y. (2016). Role of the ACE2/Angiotensin 1-7 Axis of the Renin-Angiotensin System in Heart Failure. Circ. Res..

[58] Meems L.M.G., Andersen I.A., Pan S., Harty G., Chen Y., Zheng Y., Harders G.E., Ichiki T., Heublein D.M., Iyer S.R. (2019). Design, Synthesis, and Actions of an Innovative Bispecific Designer Peptide. Hypertension.

[59] Nass R.M., Gaylinn B.D., Rogol A.D., Thorner M.O. (2010). Ghrelin and growth hormone: Story in reverse. Proc. Natl. Acad. Sci. USA.

[60] Morozumi N., Yotsumoto T., Yamaki A., Yoshikiyo K., Yoshida S., Nakamura R., Jindo T., Furuya M., Maeda H., Minamitake Y. (2019). ASB20123: A novel C-type natriuretic peptide derivative for treatment of growth failure and dwarfism. PLoS ONE.

[61] Yotsumoto T., Morozumi N., Nakamura R., Jindo T., Furuya M., Abe Y., Nishimura T., Maeda H., Ogasawara H., Minamitake Y. (2019). Safety assessment of a novel C-type natriuretic peptide derivative and the mechanism of bone- and cartilage-specific toxicity. PLoS ONE.

[62] Cataliotti A., Schirger J.A., Martin F.L., Chen H.H., McKie P.M., Boerrigter G., Costello-Boerrigter L.C., Harty G., Heublein D.M., Sandberg S.M. (2005). Oral human brain natriuretic peptide activates cyclic guanosine 3’,5’-monophosphate and decreases mean arterial pressure. Circulation.

[63] Halberg I.B., Lyby K., Wassermann K., Heise T., Zijlstra E., Plum-Morschel L. (2019). Efficacy and safety of oral basal insulin versus subcutaneous insulin glargine in type 2 diabetes: A randomised, double-blind, phase 2 trial. Lancet Diabetes Endocrinol..

[64] Januzzi J.L., Prescott M.F., Butler J., Felker G.M., Maisel A.S., McCague K., Camacho A., Pina I.L., Rocha R.A., Shah A.M. (2019). Association of Change in N-Terminal Pro-B-Type Natriuretic Peptide Following Initiation of Sacubitril-Valsartan Treatment With Cardiac Structure and Function in Patients With Heart Failure With Reduced Ejection Fraction. JAMA.

[65] Murphy S.P., Prescott M.F., Camacho A., Iyer S.R., Maisel A.S., Felker G.M., Butler J., Pina I.L., Ibrahim N.E., Abbas C. (2021). Atrial Natriuretic Peptide and Treatment With Sacubitril/Valsartan in Heart Failure With Reduced Ejection Fraction. JACC Heart Fail..

[66] Solomon S.D., McMurray J.J.V., Anand I.S., Ge J., Lam C.S.P., Maggioni A.P., Martinez F., Packer M., Pfeffer M.A., Pieske B. (2019). Angiotensin-Neprilysin Inhibition in Heart Failure with Preserved Ejection Fraction. N. Engl. J. Med..

[67] Solomon S.D., Vaduganathan M.L., Claggett B., Packer M., Zile M., Swedberg K., Rouleau J.A., Pfeffer M., Desai A., Lund L.H. (2020). Sacubitril/Valsartan Across the Spectrum of Ejection Fraction in Heart Failure. Circulation.

[68] Docherty K.F., Vaduganathan M., Solomon S.D., McMurray J.J.V. (2020). Sacubitril/Valsartan: Neprilysin Inhibition 5 Years After PARADIGM-HF. JACC Heart Fail..

[69] Iwaki T., Oyama Y., Tomoo T., Tanaka T., Okamura Y., Sugiyama M., Yamaki A., Furuya M. (2017). Discovery and dimeric approach of novel Natriuretic Peptide Receptor A (NPR-A) agonists. Bioorg. Med. Chem..

[70] Iwaki T., Nakamura Y., Tanaka T., Ogawa Y., Iwamoto O., Okamura Y., Kawase Y., Furuya M., Oyama Y., Nagayama T. (2017). Discovery and SAR of a novel series of Natriuretic Peptide Receptor-A (NPR-A) agonists. Bioorg. Med. Chem. Lett..

[71] Iwaki T., Tanaka T., Miyazaki K., Suzuki Y., Okamura Y., Yamaki A., Iwanami M., Morozumi N., Furuya M., Oyama Y. (2017). Discovery and in vivo effects of novel human natriuretic peptide receptor A (NPR-A) agonists with improved activity for rat NPR-A. Bioorg. Med. Chem..

[72] Sangaralingham S.J., Whig K., Peddibhotla S., Kirby R.J., Sessions H.E., Maloney P.R., Hershberger P.M., Mose-Yates H., Hood B.L., Vasile S. (2021). Discovery of small molecule guanylyl cyclase A receptor positive allosteric modulators. Proc. Natl. Acad. Sci. USA.

[73] Macheret F., Boerrigter G., McKie P., Costello-Boerrigter L., Lahr B., Heublein D., Sandberg S., Ikeda Y., Cataliotti A., Bailey K. (2011). Pro-B-type natriuretic peptide(1-108) circulates in the general community: Plasma determinants and detection of left ventricular dysfunction. J. Am. Coll. Cardiol..

[74] Buglioni A., Cannone V., Cataliotti A., Sangaralingham S.J., Heublein D.M., Scott C.G., Bailey K.R., Rodeheffer R.J., Dessi-Fulgheri P., Sarzani R. (2015). Circulating aldosterone and natriuretic peptides in the general community: Relationship to cardiorenal and metabolic disease. Hypertension.

[75] Asferg C.L., Nielsen S.J., Andersen U.B., Linneberg A., Moller D.V., Hedley P.L., Christiansen M., Goetze J.P., Esler M., Jeppesen J.L. (2013). Relative atrial natriuretic peptide deficiency and inadequate renin and angiotensin II suppression in obese hypertensive men. Hypertension.

[76] Reginauld S.H., Cannone V., Iyer S., Scott C., Bailey K., Schaefer J., Chen Y., Sangaralingham S.J., Burnett J.C. (2019). Differential Regulation of ANP and BNP in Acute Decompensated Heart Failure: Deficiency of ANP. JACC Heart Fail..

[77] Bachmann K.N., Gupta D.K., Xu M., Brittain E., Farber-Eger E., Arora P., Collins S., Wells Q.S., Wang T.J. (2021). Unexpectedly Low Natriuretic Peptide Levels in Patients With Heart Failure. JACC Heart Fail..

[78] DiMasi J.A., Grabowski H.G., Hansen R.W. (2016). Innovation in the pharmaceutical industry: New estimates of R&D costs. J. Health Econ..

